# Gαs is dispensable for β-arrestin coupling but dictates GRK selectivity and is predominant for gene expression regulation by β2-adrenergic receptor

**DOI:** 10.1016/j.jbc.2023.105293

**Published:** 2023-09-27

**Authors:** Valeria Burghi, Justine S. Paradis, Adam Officer, Sendi Rafael Adame-Garcia, Xingyu Wu, Edda S.F. Matthees, Benjamin Barsi-Rhyne, Dana J. Ramms, Lauren Clubb, Monica Acosta, Pablo Tamayo, Michel Bouvier, Asuka Inoue, Mark von Zastrow, Carsten Hoffmann, J. Silvio Gutkind

**Affiliations:** 1 Moores Cancer Center, University of California San Diego, La Jolla, California, USA; 2 Department of Pharmacology, University of California San Diego, La Jolla, California, USA; 3 Institut für Molekulare Zellbiologie, CMB - Center for Molecular Biomedicine, Universitätsklinikum Jena, Friedrich-Schiller-Universität Jena, Jena, Germany; 4 Department of Pharmaceutical Chemistry, University of California San Francisco, San Francisco, California, USA; 5 Department of Biochemistry and Molecular Medicine, Institute for Research in Immunology and Cancer, Université de Montréal, Québec, Canada; 6 Graduate School of Pharmaceutical Sciences, Tohoku University, Sendai, Miyagi, Japan; 7 Department of Psychiatry and Department of Cellular and Molecular Pharmacology, University of California San Francisco, San Francisco, California, USA

**Keywords:** G protein–coupled receptor, G protein, β-arrestin, signaling, gene expression

## Abstract

β-arrestins play a key role in G protein–coupled receptor (GPCR) internalization, trafficking, and signaling. Whether β-arrestins act independently of G protein–mediated signaling has not been fully elucidated. Studies using genome-editing approaches revealed that whereas G proteins are essential for mitogen-activated protein kinase activation by GPCRs., β-arrestins play a more prominent role in signal compartmentalization. However, in the absence of G proteins, GPCRs may not activate β-arrestins, thereby limiting the ability to distinguish G protein from β-arrestin-mediated signaling events. We used β2-adrenergic receptor (β2AR) and its β2AR-C tail mutant expressed in human embryonic kidney 293 cells wildtype or CRISPR–Cas9 gene edited for Gα_s_, β-arrestin1/2, or GPCR kinases 2/3/5/6 in combination with arrestin conformational sensors to elucidate the interplay between Gα_s_ and β-arrestins in controlling gene expression. We found that Gα_s_ is not required for β2AR and β-arrestin conformational changes, β-arrestin recruitment, and receptor internalization, but that Gα_s_ dictates the GPCR kinase isoforms involved in β-arrestin recruitment. By RNA-Seq analysis, we found that protein kinase A and mitogen-activated protein kinase gene signatures were activated by stimulation of β2AR in wildtype and β-arrestin1/2-KO cells but absent in Gα_s_-KO cells. These results were validated by re-expressing Gα_s_ in the corresponding KO cells and silencing β-arrestins in wildtype cells. These findings were extended to cellular systems expressing endogenous levels of β2AR. Overall, our results support that Gs is essential for β2AR-promoted protein kinase A and mitogen-activated protein kinase gene expression signatures, whereas β-arrestins initiate signaling events modulating Gα_s_-driven nuclear transcriptional activity.

With more than 800 coding genes in the human genome, G protein–coupled receptors (GPCRs) represent the largest family of cell surface proteins involved in signal transduction (1). By serving as receptors for a wide range of ligands, GPCRs play key roles in many physiological processes, and their dysfunction contributes to some of the most prevalent human diseases (2, 3). GPCRs have immense therapeutic potential, as ∼34% of all drugs approved by the US Food and Drug Administration target 108 GPCRs or their related signaling pathways (4, 5).

The classical GPCR signaling view for long has been that upon ligand binding the receptor undergoes conformational changes leading to its association with the Gα subunit of the heterotrimeric G protein, thereby promoting the release of GDP and its exchange for GTP (6). While GTP-Gα initiates signal transmission, G protein–coupled receptor kinases (GRKs) phosphorylate the C-terminal tail of the receptor (7). This leads to recruitment of β-arrestins, which causes receptor desensitization by uncoupling receptors from G proteins and promotes their internalization through clathrin-coated pits, resulting in G protein signal termination at the plasma membrane (PM) (2). The endocytosis then contributes to resensitization through receptor dephosphorylation and recycling to the PM (8). More recently, long-lasting G protein–mediated signaling in the endosomes has been documented (9). Numerous reports have also described G protein–independent roles of β-arrestins in GPCR signal transduction, thereby challenging this canonical paradigm (10). Among them, the activation of the extracellular signal–regulated kinase 1/2 (ERK 1/2, collectively referred here as ERK) cascade represents the earliest and likely most prominent example (11, 12, 13, 14).

Using genome-editing approaches, our team and others have explored the relative contribution of G proteins and β-arrestins to the overall ERK activation triggered by GPCRs, including the β2-adrenergic receptor (β2AR) (15, 16, 17, 18). β2AR is one of the most extensively characterized Gα_s_-coupled receptors, which has served as a model to elucidate the fundamental mechanisms by which GPCRs control intracellular signaling in key physiological functions and pathological conditions (19, 20, 21). However, G protein–independent signaling by β2AR has not been fully investigated, raising the possibility that reduced expression of Gα_s_ may hamper the ability of these receptors to recruit and activate β-arrestins, and that in turn this may contribute to the overall signaling deficiency in the absence of Gα_s_. Furthermore, the role of nuclear signaling by β2AR through β-arrestins in the absence of a functional Gα_s_ protein has not been evaluated. This has prevented a comprehensive understanding of the relative contribution of Gα_s_ and β-arrestins to the overall β2AR signaling, thereby limiting our appreciation of how β-arrestin- or Gα_s-_biased ligands may affect their therapeutic outcomes.

## Results

### Gα_s_ is dispensable for β2AR internalization and β-arrestin2 recruitment

**Figure 1:**
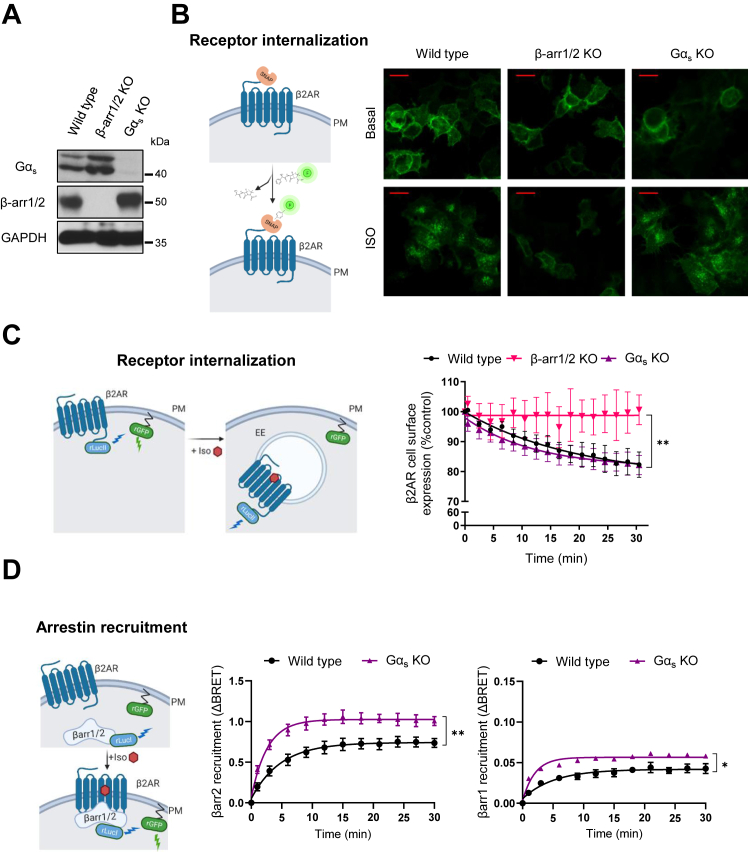
**β2AR internalization and β-arrestin recruitment in the absence of Gα**_**s**_**protein.***A*, Western blot for Gα_s_ protein and β-arrestin1/2 in HEK293 wildtype, β-arrestin1/2 KO, and Gα_s_ KO cells. GAPDH was used as loading control. *B*, scheme of SNAP-tagged β2AR used for immunofluorescence (IF) imaging and confocal fluorescence microscopy of HEK293 wildtype and their derived cells transiently expressing this receptor. *Top panels*: basal condition; *bottom panels*: following a 15 min stimulation with 1 μM isoproterenol (ISO). Data are representative of three independent experiments. Scale bars represent 20 μm. *C* and *D*, illustration of the EbBRET assays used to monitor β2AR and β-arrestin1/2 trafficking (*left panels*) and corresponding time-dependent assessments (*right panels*). Cells were incubated in the absence or the presence of 1 μM ISO, and BRET measurements were obtained as described in Experimental procedures section. *C*, HEK293 wildtype and their derived cells were transfected with HA-β2AR, β2AR-RLucII, and rGFP-CAAX plasmids. The BRET changes on ISO treatment are expressed as a percentage of BRET change observed in the control (vehicle) condition. Data represent the mean ± SEM of three independent experiments. *D*, HEK293 wildtype and Gα_s_ KO cells were transfected with HA-β2AR, RLucI-β-arrestin, and rGFP-CAAX plasmids. The BRET changes on ISO treatment represent the mean ± SEM of three independent experiments. β2AR, β2-adrenergic receptor; EbBRET, nhanced bystander bioluminescence energy transfer; HA, hemagglutinin; HEK293, human embryonic kidney 293 cell line.

We initiated the study of G protein–independent signaling by β2AR addressing the role of Gα_s_ protein in receptor internalization. To do so, we took advantage of human embryonic kidney 293 (HEK293) cells depleted by CRISPR–Cas9 technology of Gα_s_ (Gα_s_ KO) (22) or β-arrestin1 and β-arrestin2 (β-arr1/2 KO) (15). We confirmed loss of expression of Gα_s_ and β-arrestin1 and β-arrestin2 in the corresponding cells (Fig. 1*A*) and surface β2AR abundance after transfection (Fig. S1). We visualized receptor internalization using the SNAP-tag system that specifically labels cell surface–expressed β2AR (Fig. 1*B*). HEK293 wildtype cells internalized SNAP-β2AR following isoproterenol stimulation. Aligned with previous results from our team (15) and others (16, 18), β-arr1/2 KO cells did not internalize β2AR. However, isoproterenol-induced internalization was conserved in Gα_s_ KO cells. As a complementary approach, we used enhanced bystander bioluminescence energy transfer (EbBRET) sensors that allow quantitative monitoring of GPCR trafficking (23). In this case, we used the BRET acceptor GFP from *Renilla reniformis* (rGFP) expressed at the PM partnered with the mutant form of Rluc, RlucII, as the donor fused to β2AR. For targeting to PM, rGFP was fused through its C terminus to the polybasic sequence and prenylation CAAX box of KRas (rGFP-CAAX) (Fig. 1*C*, *left panel*). Coexpression of β2AR-RlucII and rGFP-CAAX produced detectable basal EbBRET signal, because of their colocalization and enrichment at the PM, favoring bystander encounter between the donor and acceptor. In agreement with the confocal images, in wildtype cells, isoproterenol mediated receptor internalization, by decreasing the donor:acceptor ratio at the PM and reducing the EbBRET signal in a time-dependent manner. Gα_s_ absence did not affect β2AR internalization, but no such loss of PM receptor was observed in βarr1/2 KO cells indicating that internalization was β-arrestin dependent and did not require Gα_s_ (Fig. 1*C*, *right panel*).

We next assessed whether β-arrestins are recruited to the PM upon β2AR activation in the absence of Gα_s_ protein. We used RLuc as the donor fused to β-arrestin1 or β-arrestin2 and rGFP-CAAX as the acceptor (Fig. 1*D*, *left panel*). As predicted, isoproterenol stimulation of wildtype cells expressing β2AR robustly increased the BRET signal between RLucI-β-arrestin2 and rGFP-CAAX, whereas a more modest response was observed for RLucI-β-arrestin1 (Fig. 1*D*, *right panel*), confirming β2AR coupling preference for β-arrestin2, as previously described for class A GPCRs (24, 25). Remarkably, β-arrestin recruitment to the PM upon β2AR activation was conserved in Gα_s_-KO cells and even slightly increased. Altogether, these data indicate that β-arrestin recruitment by β2AR and receptor internalization occur independently of Gα_s_ protein signaling.

### Gα_s_ is not required for isoproterenol-induced β2AR and β-arrestin2 conformational changes in living cells

**Figure 2:**
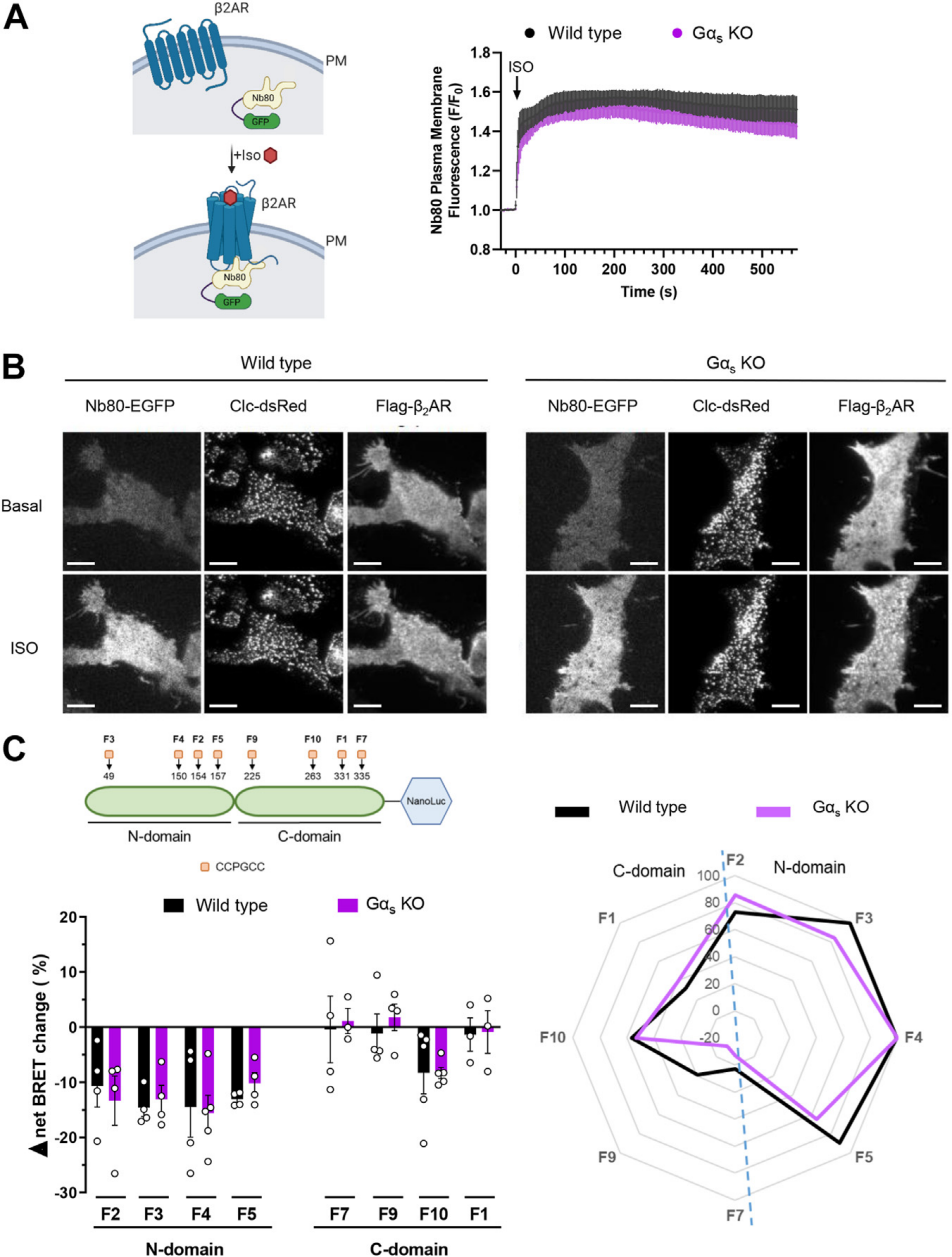
**β2AR and β-arrestin2 conformational changes in the absence of Gα**_**s**_**protein.***A* and *B*, Scheme for detecting β2AR conformational activation with Nb80–GFP and plasma membrane fluorescence monitored by live-cell TIRF microscopy. HEK293 wildtype, and Gα_s_ KO cells were transfected with FLAG-β2AR, peGFP-N1 Nb80, and clathrin light chain–dsRed plasmids. Cells were treated with 10 μM ISO at time 0 for a time course. *A*, Nb80–GFP plasma membrane fluorescence measurements for 10 min and quantitative image analysis were performed as described in the Experimental procedures section. Data represent the mean ± SEM of three independent experiments. *B*, TIRF microscopy frames showing Nb80–GFP, clathrin light chain–dsRed, and FLAG-β2AR before (*top panels*) and after ISO addition (*bottom panels*). Representative of three independent experiments. Scale bars represent 10 μm. *C*, schematic representation of the intramolecular β-arrestin2-FlAsH-Nanoluciferase (NanoLuc) biosensors (*left top panel*) and β-arrestin2 conformational changes upon β2AR activation (*left bottom* and *right panels*). The NanoLuc BRET donor was genetically fused to the β-arrestin2 C terminus, and the FlAsH-binding motif (CCPGCC) was introduced at eight different positions in the N and C domains (F1–F5, F7, F9, F10, and amino acid sequence positions denoted). HEK293 wildtype and Gα_s_ KO cells were transfected with untagged β2AR and each β-arrestin2 biosensor plasmid, FlAsH-labeled and stimulated with different concentrations of ISO, ranging from 1 nM to 10 μM. BRET measurements were obtained as described in the Experimental procedures section. For the bar graph and radar plot, the values of the highest concentrations (1 and 10 μM) were used. Bar graph shows BRET change in percentage and represents mean ± SEM of at least three independent experiments. Radar chart shows mean BRET changes of at least three independent experiments normalized to the F4 biosensor values, as this sensor shows maximum conformational change. HEK293, human embryonic kidney 293 cell line; ISO, isoproterenol; TIRF, total internal reflection fluorescence.

β2AR has served as a useful model system to elucidate, through structural and biophysical studies, the mechanism of activation of GPCRs (26). The current proposed model for β2AR activation suggests that unliganded β2AR exists as ensembles of discrete conformations in dynamic equilibrium, and that agonist binding increases β2AR conformational dynamics between intermediate and active states (19). In purified systems, the latter are stabilized in the presence of Gα_s_ protein or active state–stabilizing chaperones such as nanobodies (20, 26, 27, 28). Overexpression of either Gα_s_ or β-arrestin2 potentiates the isoproterenol-promoted β2AR conformational changes observed with a BRET-based unimolecular receptor conformational sensor, and these changes could be observed in cells lacking β-arrestins or Gαs, thus suggesting that either effectors can stabilize an active state of the receptor (29). To further explore the roles of each effector in the stabilization of receptor-active states, we used here live cell imaging and a biosensor that detects activated β2AR based on a conformation-specific single-domain camelid antibody (Nb80) fused to enhanced GFP (Nb80–GFP) (30) (Fig. 2*A*, *left panel*). Through total internal reflection fluorescence (TIRF) microscopy, we monitored PM fluorescence in wildtype and Gα_s_ KO cells (Fig. 2, *A* and *B*). Analysis of basal condition showed the absence of Nb80–GFP fluorescence at the PM for both cell types and revealed that after incubation with isoproterenol, Nb80–GFP was rapidly recruited to the PM to the same extent in wildtype and Gα_s_ KO cells (Fig. 2, *A* and *B*). We verified PM distribution of the expressed β2AR and separation of Nb80–GFP localization from that of clathrin light chain (Fig. 2*B*), thus reflecting the first phase of Nb80–GFP recruitment prior to its second phase association with activated β2AR in the endosomes (30). These results confirm that the absence of Gα_s_ protein does not affect the isoproterenol-induced β2AR conformational changes at the PM revealed by Nb80–GFP recruitment.

However, the possibility still exists that in the absence of Gα_s_, β2AR recruits β-arrestins but may not promote β-arrestin conformational changes reflecting its activation. Several β-arrestin conformational sensors are currently available (31, 32, 33, 34). Here, we focused on β-arrestin2 and took advantage of our recently described panel of intramolecular β-arrestin2–fluorescein arsenical hairpin (FlAsH)–Nanoluciferase (NanoLuc) biosensors (34) (Fig. 2*C*, *left top panel*). In this case, each biosensor has a NanoLuc BRET donor genetically fused to the β-arrestin2 C terminus and a FlAsH binding motif, CCPGCC, introduced at eight different positions in the N and C domains (F1–F5, F7, F9, and F10). The biosensors display reduction of intramolecular BRET between the NanoLuc donor and the FlAsH acceptor to report on receptor-induced β-arrestin2 conformational changes. We performed concentration–response curves for isoproterenol in HEK293 wildtype and Gα_s_ KO cells transfected with β2AR and one β-arrestin2 biosensor. The mean Δnet BRET changes at saturating ligand concentration are depicted as bar charts for both cell types separated into FlAsH sensors located in the N (F2–F5) and C (F1, F7, F9, and F10) domains (Fig. 2*C*, *left bottom panel*). Aligned with previous results using FRET-based biosensors with β2AR (32), in wildtype cells, we obtained the greatest β-arrestin2 conformational differences with the N-domain biosensors (F2–F5), concomitant with a reduction of BRET signal for one C-domain sensor (F10). These studies revealed a similar fingerprint of β-arrestin2 conformational changes in Gα_s_ KO compared with wildtype cells (Fig. 2*C*, *right panel*). Altogether, these data suggest that Gα_s_ protein is not necessary for the isoproterenol-induced β2AR and the β-arrestin2 conformational changes in living cells.

### Gα_s_ protein dictates the GRK isoforms involved in β-arrestin recruitment after β2AR stimulation

**Figure 3:**
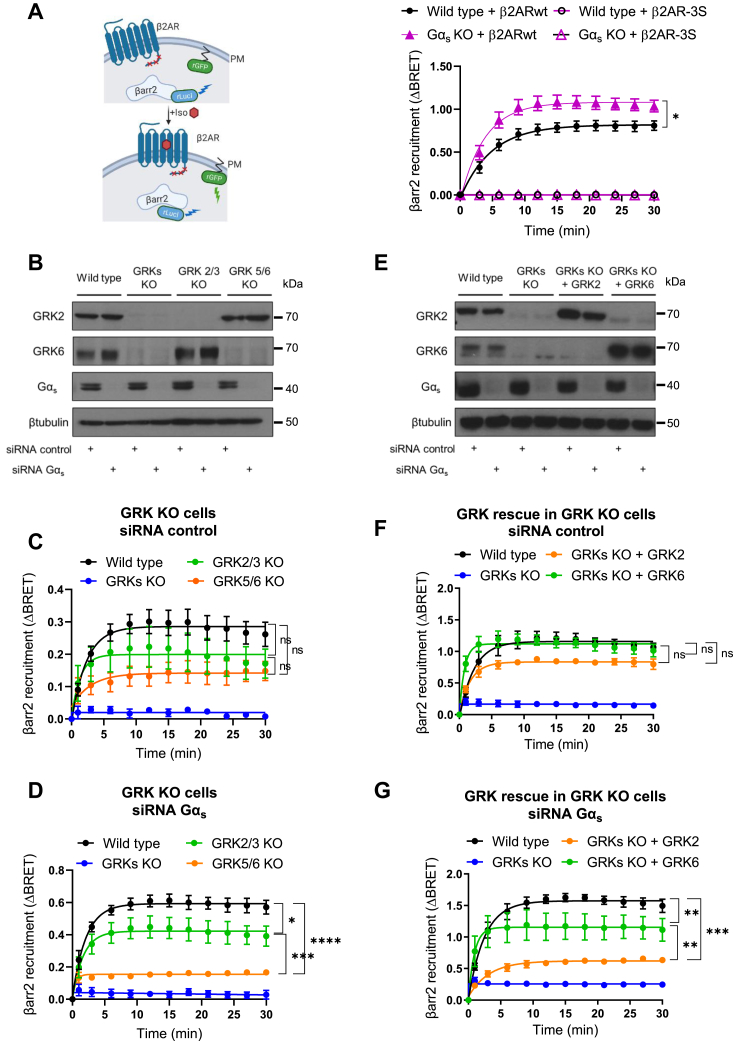
**Role of β2AR C-terminal tail phosphorylation and GRKs isoforms in β-arrestin2 recruitment.** For all β-arrestin2 recruitment assays, the cells were transfected with the RLucI-β-arrestin2-rGFP-CAAX donor–acceptor pair and incubated in the absence or the presence of 1 μM ISO. BRET measurements were obtained for 30 min as described in the Experimental procedures section. *A*, illustration of the EbBRET assay used to monitor β-arrestin2 trafficking and corresponding time-dependent assessment in HEK293 wildtype and Gα_s_ KO cells transfected with β2AR wildtype or a phosphorylation-deficient mutant β2AR-3S. *B*–*D*, HEK293 wildtype, GRK2/3 KO, GRK5/6 KO, and GRK2/3/5/6 (GRKs) KO cells were first transfected with *GNAS* and control siRNAs for 24 h, followed by medium change before transfection with HA-β2AR and the donor–acceptor pair for another 48 h. *E*–*G*, HEK293 wildtype and GRK KO cells were first transfected with *GNAS* and control siRNAs for 24 h, followed by medium change before transfection for another 48 h with HA-β2AR, the donor–acceptor pair, and GRK2 or GRK6 plasmids (where indicated). *B* and *E*, Western blot showing GRK2, GRK6, and Gα_s_ expression. β-tubulin was used as loading control. Blots in (*E*) correspond to a longer time of exposure compared with (*B*), which may have caused visualization of very slight bands in the GRK2 KO lanes, an apparent doublet of GRK6 and qualitatively different banding pattern of GRK6 and Gα_s_. *A*, *C*, *D*, *F*, and *G*, the BRET changes on ISO treatment represent the mean ± SEM of three independent experiments. β2AR, β2-adrenergic receptor; BRET, bioluminescence energy transfer; EbBRET, enhanced bystander BRET; GRK, G protein–coupled receptor kinase; HA, hemagglutinin; HEK293, human embryonic kidney 293 cell line; ISO, isoproterenol.

Upon activation, conformational changes in β2AR lead to its C-tail phosphorylation, including residues S355, S356, T360, and S364 by GRKs, which subsequently promote β-arrestin recruitment and its functional activation (35). To explore if coupling to Gα_s_ affects this process, we first confirmed that a β2AR mutant lacking the three serine C-terminal GRK phosphorylation sites (S355G, S356G, and S364G) does not recruit β-arrestin2 (30, 36) (Fig. 3*A*). We found that this strict requirement for these phosphor-acceptor sites in β2AR was maintained in Gα_s_-KO cells (Fig. 3*A*). We next took advantage of our recently developed panel of GRK KO cells lacking GRK2/3 or GRK5/6 and quadruple GRK2/3/5/6 (GRK) KO cells (37). The absence of these GRKs was confirmed by Western blotting in the respective genome-edited cells (Fig. 3*B*). In cells expressing Gα_s_, the recruitment of β-arrestin2 was abolished by complete GRK KO but maintained in cells expressing only GRK2/3 or GRK5/6, albeit GRK5/6 KO cells showed a slightly smaller recruitment (Fig. 3*C*). This suggests that these two families of GRKs can on their own support β-arrestin recruitment, with a potentially more prevalent role for GRK5/6 in wildtype cells. However, when Gα_s_ was knocked down, the two GRK families played distinct functional roles, with GRK5/6 being nearly essential and GRK2/3 partially required (Fig. 3*D*). As an orthogonal approach, we performed rescue studies re-expressing GRK2 and GRK6, as representative subfamily members, in GRK KO cells (Fig. 3*E*). Either GRK6 or GRK2 could rescue β-arrestin2 recruitment in cells expressing Gα_s_ (Fig. 3*F*), but only GRK6 rescued the membrane β-arrestin2 BRET signal in Gα_s_ knockdown cells after isoproterenol stimulation of β2AR (Fig. 3*G*). Although we cannot rule out that this could be partially affected by a higher expression of transfected GRK6 as compared with GRK2, aligned with our prior studies (38), the overall findings suggest that Gα_s_ coupling may dictate the specificity of GRKs involved in β-arrestin2 recruitment after β2AR stimulation. While both GRKs contribute to this process in wildtype cells, in the absence of Gα_s,_ the GRK5/6 subfamily plays a primary role.

**Figure 4:**
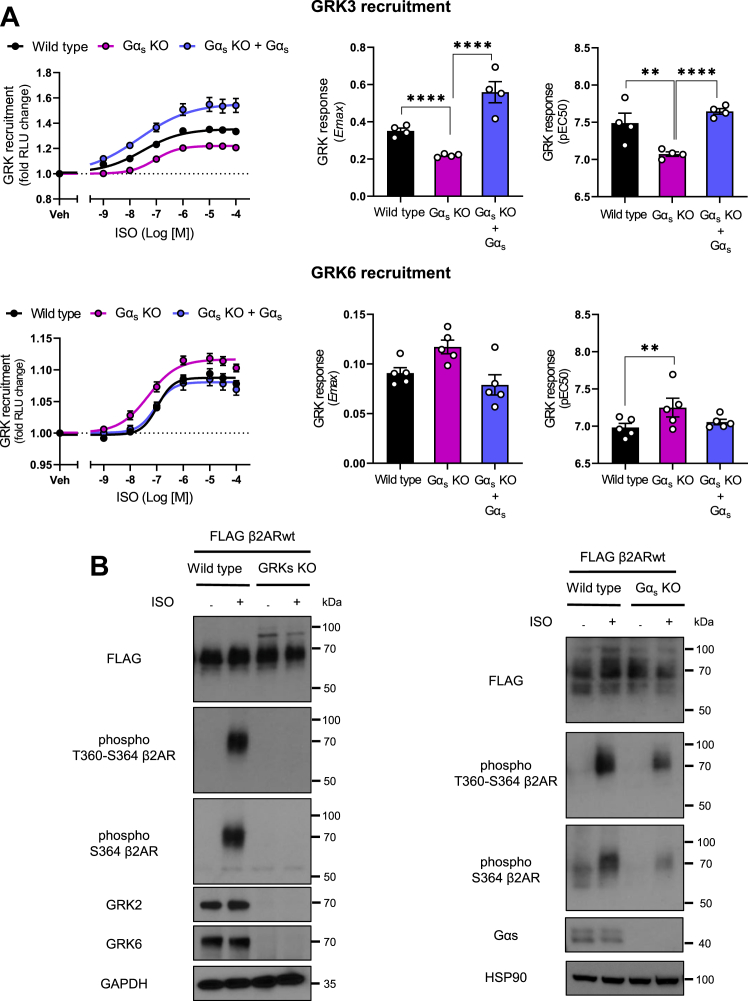
**GRK isoforms recruitment to the β2AR and receptor phosphorylation in the absence of Gα**_**s**_**.***A*, concentration–response curves and parameters of the NanoBit-GRK recruitment assay. β2AR-SmBiT was expressed together with the indicated GRK-LgBiT constructs in wildtype and Gα_s_ KO cells. For the rescue condition, Gα_s_ KO cells were also cotransfected with the human Gα_s_ short isoform plasmid. Cells were stimulated with different concentrations of ISO, and luminescence measurements were obtained as described in the Experimental procedures section. The GRK recruitment signals were fitted to a concentration–response curve to estimate the Emax and pEC_50_ per condition. The data represent the mean ± SEM of at least four independent experiments. *B*, detection of β2AR phosphorylation with phosphosite-specific antibodies against pT360/S364 and pS364 in wildtype, GRK2/3/5/6 (GRKs) KO, and Gα_s_ KO cells. Before immunoprecipitation, cells stably expressing FLAG-tagged β2AR were stimulated with 10 μM ISO or vehicle for 10 min. Blots are representative of three independent experiments. β2AR, β2-adrenergic receptor; GRK, G protein–coupled receptor kinase; ISO, isoproterenol.

To address this possibility, we performed direct measurements of GRK recruitment to β2AR upon isoproterenol stimulation in wildtype and Gα_s_-KO cells (Fig. 4*A*). For these studies using the NanoBiT-GRK recruitment assay (37), we chose GRK3 and GRK6 as representative subfamily members because they display the largest signal dynamic range (37). Gα_s_ KO cells showed lower levels of GRK3 recruitment to β2AR when compared with wildtype cells, indicated by the corresponding Emax and pEC_50_ values (Fig. 4*A*, *top panels*). Importantly, this effect was rescued after re-expressing Gα_s_ in the KO cells. In contrast, Gα_s_ KO cells showed a trend of higher GRK6 recruitment to β2AR compared with wildtype cells and reversion of this effect after re-expressing Gα_s_ (Fig. 4*A*, *bottom panels*). Thus, these findings reinforce the idea that Gα_s_ protein determines which GRKs are recruited after β2AR stimulation, namely the absence of Gα_s_ limits GRK2/3 recruitment and activity.

To further address the limited role of GRK2/3 in the absence of Gα_s_, we studied the status of β2AR phosphorylation using phosphosite-specific antibodies (Fig. 4*B*), directed against β2AR pT360-S364 or pS364 alone, which represent preferential GRK2 sites (35). We observed phosphorylation of β2AR T360 and S364 sites in wildtype cells after isoproterenol stimulation, and this response was blunted in GRK KO cells (Fig. 4*B*, *left*). In Gα_s_ KO cells, isoproterenol led to lower levels of β2AR pT360 and pS364 compared with wildtype cells (Fig. 4*B*, *right panel*), further supporting that the absence of Gα_s_ leads to impaired GRK2/3 subfamily activity.

### β2AR nuclear transcriptional responses in the absence of βarrestin1/2 and Gα_s_ protein

**Figure 5:**
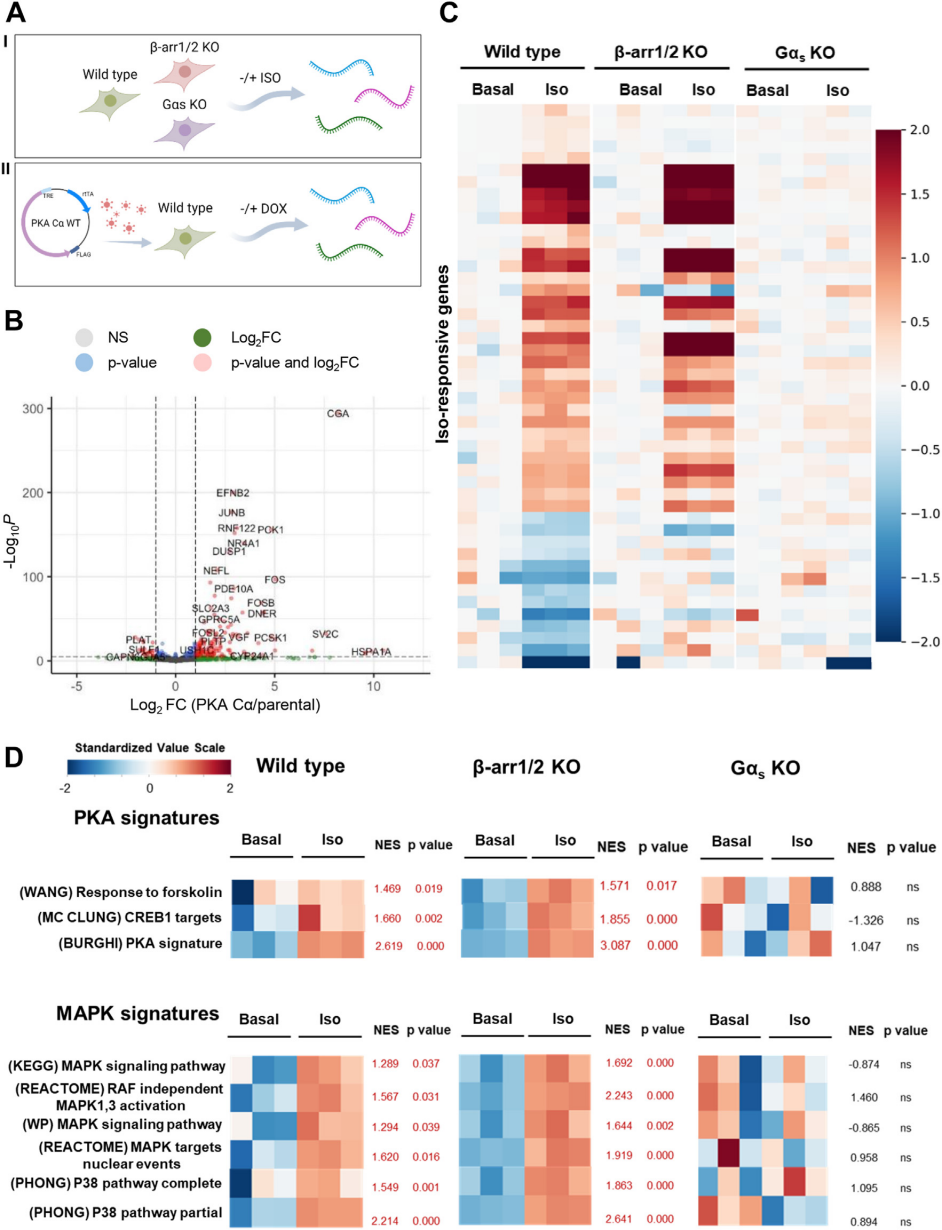
**β2AR transcriptional response in the absence of βarrestin1/2 and Gα**_**s**_**protein.***A*, scheme of the procedures to obtain (I) the β2AR transcriptional response and (II) the PKA signature. *B*, volcano plot highlighting key differentially expressed genes in HEK293 PKA Cα cells compared with HEK293 parental cells, both treated with doxycycline. Our PKA signature consists of the top 100 upregulated genes. *C*, heat map depicting the transcript expression profiles in HEK293 cells transfected with β2AR, wildtype cells, β-arrestin1/2 KO, and Gα_s_ KO cells, under control condition and stimulated with ISO for 1 h. The rows correspond to differentially expressed genes in wildtype cells stimulated with ISO compared with basal condition. Levels of gene expression are indicated on the color scale on the *right*. The values are normalized rLog from DESeq2 further normalized to the median of the intracell type basal condition. *D*, heat maps showing enrichment profiles for gene sets related to PKA and MAPK pathways from the Molecular Signatures Database (MSigDB), c2 collection, computed using gene set enrichment analysis (GSEA). Our in-house PKA signature was included in the analysis. Levels of gene set enrichment are indicated on the standardized value scale on the *top left*. Normalized enriched score (NES) and *p* values are indicated; in *red*, *p* < 0.05; ns, not significant. β2AR, β2-adrenergic receptor; HEK293, human embryonic kidney 293 cell line; ISO, isoproterenol; MAPK, mitogen-activated protein kinase.

While the activation of second messenger–generating systems explains most rapid physiological and pharmacological responses elicited by GPCRs, prolonged stimulation leads to nuclear transcriptional responses affecting cellular behaviors and states, including normal and aberrant cell growth (39). In this regard, regulation of gene expression often involves the signals regulated by second messenger–regulated kinases, such as protein kinase A (PKA) acting downstream from Gs and cAMP accumulation, and the large family of ERKs, among which ERK1/2 are the prototypes (40, 41). Ultimately, these signals converge on the regulation of transcription factors that control gene expression (42, 43). To explore the role of Gα_s_ and β-arrestin in nuclear transcriptional programs in a more global unbiased approach, we performed RNA-Seq studies in cells lacking either of these two signaling arms downstream from β2AR (Fig. 5*A*), followed by detailed bioinformatics analysis of gene expression signatures. In this process, we noticed a dearth of information on PKA-regulated gene sets. Thus, we first used the tetracycline-regulated expression of wildtype PKA Cα subunit in HEK293 cells (Fig. 5*A*) to develop a PKA signature in the same cellular context (Fig. 5*B*). This approach revealed multiple PKA-regulated genes, including well-known PKA downstream transcriptional targets, such as PCK1 and FOS (44), and many new PKA transcriptional targets whose underlying mechanism can now be explored (Fig. 5*B* and Table S1). As shown in Figure 5*C*, we noticed multiple genes whose individual expression levels were distinct in β-arrestin1/2 and Gα_s_ KO cells. Rather than focusing on the individual gene level, we performed a detailed analysis of gene signatures taking advantage of large datasets that were recently compiled as part of our Molecular Signatures Database (MSigDB) (Tables S2–S4). This approach supported that β-arrestins are not required for the activation of PKA gene signatures, including prior datasets of forskolin targets and CREB1 targets and our more specific PKA signature, whereas Gα_s_ is essential (Fig. 5*D*). β2AR activation also led to significant changes in multiple mitogen-activated protein kinase (MAPK) signatures, which clearly support the activation of MAPK nuclear transcriptional programs by these receptors. Although individual variations do exist reflecting the complexities of β-arrestin- and Gα_s_-mediated signaling events, stimulation of these MAPK-regulated gene signatures was not significantly reduced in β-arrestin1/2 KO cells but abolished in Gα_s_ KO cells (Fig. 5*D*).

**Figure 6:**
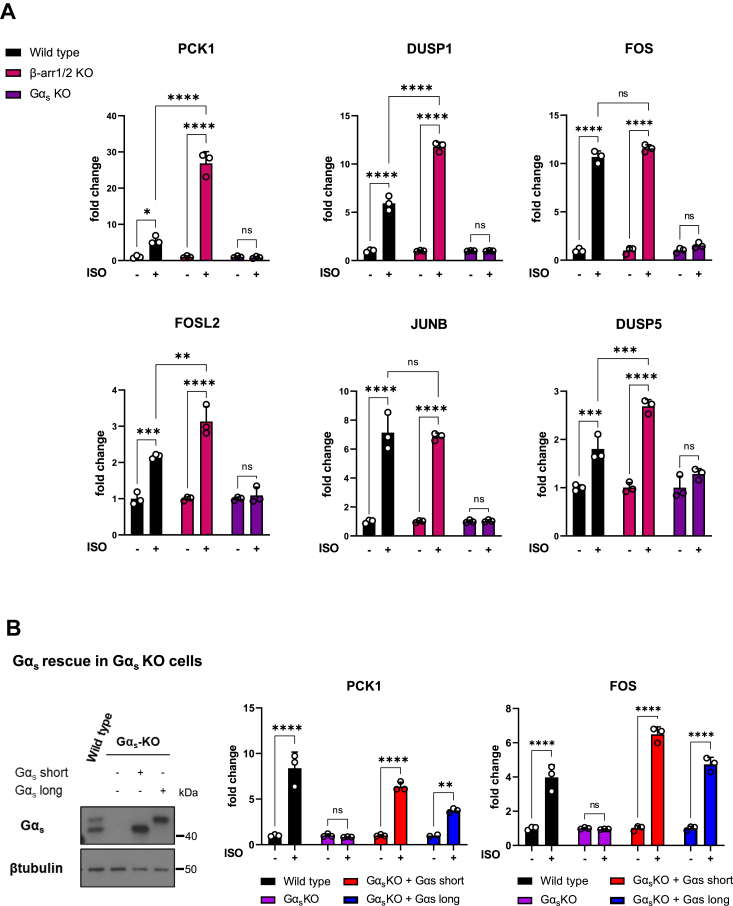
**Expression profiles of β2AR target genes in the absence of βarrestin1/2 and Gα**_**s**_**protein.***A*, mRNA levels of representative PKA and MAPK signature genes from RNA-Seq data of HEK293 wildtype, β-arrestin1/2 KO, and Gα_s_ KO cells upon ISO stimulation. The data represent the mean ± SD, n = 3. *B*, Gα_s_ rescue of PCK1 and FOS gene expression in Gα_s_ KO cells. Cells were transfected with HA-β2AR and Gα_s_ protein (short or long isoforms) plasmids. HEK293 wildtype were used as controls. Cells were incubated with 1 mM ISO or vehicle for 1 h, and mRNA levels of the indicated genes were determined by qPCR as described in the Experimental procedures section. *Left panel*: Western blot showing Gα_s_ expression. β-tubulin was used as loading control. The data represent the mean ± SD of three independent experiments. β2AR, β2-adrenergic receptor; HA, hemagglutinin; HEK293, human embryonic kidney 293 cell line; ISO, isoproterenol; MAPK, mitogen-activated protein kinase; qPCR, quantitative PCR.

These findings were further extended by the analysis of specific representative genes, including PCK1, DUSP1, FOS, FOSL2, JUNB, and DUSP5 (Fig. 6*A*). In every case, KO of β-arrestin1/2 did not diminish the expression of these genes, which in some cases were instead significantly increased. This may reflect the dual action of β-arrestins, the desensitization of the Gs pathway, and their own signaling activity. Gα_s_ KO abolished their responses, and this Gα_s_ dependence was confirmed by rescuing experiments re-expressing Gα_s_ in Gα_s_ KO cells, using PCK1 and FOS as examples (Fig. 6*B*). In this case, we confirmed that both short and long forms of Gα_s_ can rescue Gα_s_ deficiency when re-expressed at similar levels. The absence of PKA and MAPK gene signatures regulation in Gα_s_ KO cells together with the latter rescue experiments support the predominant role of Gα_s_ protein in nuclear signaling by β2AR.

In this regard, in previous studies, we have evaluated a potential role for Gα_i_ protein in β2AR signaling to ERK and found that Gα_i_ inhibition with pertussis toxin (PTX) failed to prevent ERK activation (15). Here, we took advantage of cells stably expressing Gα_s-_ or Gα_i_-coupled designer receptors that are exclusively activated by designer drugs (DREADDs), as controls (45). As expected, while pretreatment of Gα_s_-DREADD cells with PTX did not affect ERK activation upon clozapine *N*-oxide (CNO) stimulation, it abolished ERK response in Gα_i_-DREADD cells (Fig. S2, *left panel*). Under the same conditions, PTX did not impair isoproterenol-induced ERK phosphorylation in HEK293 wildtype cells expressing β2AR (Fig. S2, *right panel*). These results provide further support that Gα_i_ signaling does not play a major role in ERK activation by β2AR.

Finally, the lack of β-arrestin requirement for the expression of representative β2AR target genes was confirmed using HEK293 cells in which β-arrestin1/2 cells were transiently knocked down (Fig. S3*A*). The fact that the absence of β2AR internalization does not restrict gene expression regulation was also evaluated using the C-tail phosphoacceptor mutant β2AR that does not recruit β-arrestins and yet stimulated the expression of representative genes equal or more than β2AR wildtype (Fig. S3*B*).

### Roles for Gα_s_ protein and β-arrestins in ERK activation and gene expression by endogenously expressed β2AR

**Figure 7:**
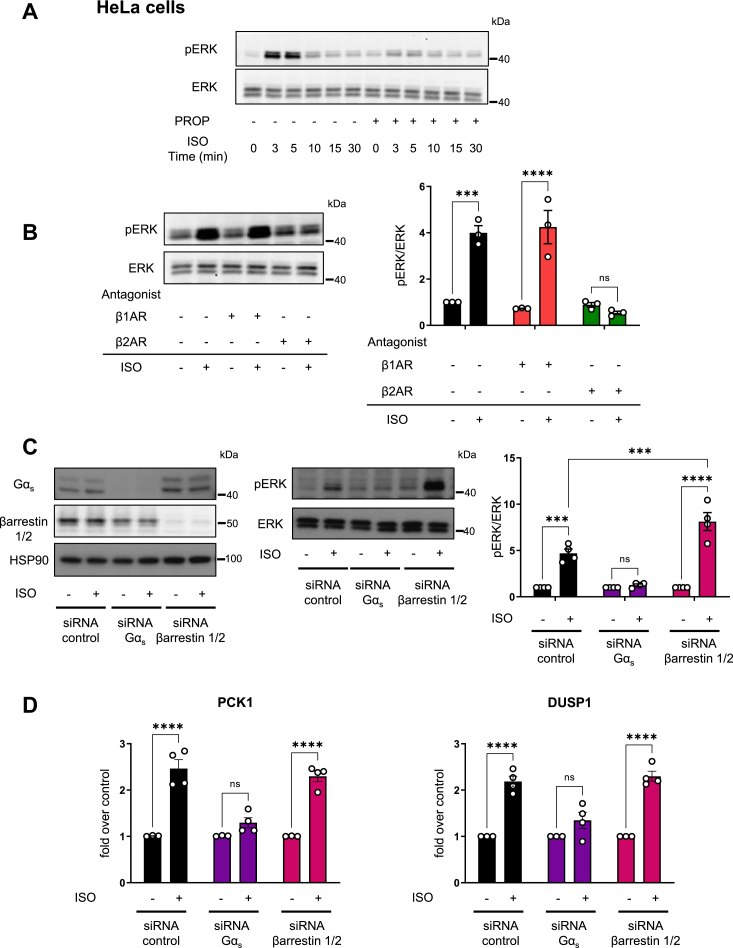
**ERK activation and expression levels of β2AR target genes in HeLa cells in the absence of Gα**_**s**_**or β-arrestin1/2.***A*, time course of ERK activation. HeLa cells were pretreated with 10 μM propranolol (PROP) for 30 min and stimulated with 10 μM ISO for different period (0–30 min). *B*, ERK activation in the presence of β1AR and β2AR selective antagonists. HeLa cells were pretreated with 300 nM of β1AR (CGP 20712) or β2AR antagonists (ICI 118,551) for 30 min and stimulated with 1 μM ISO or vehicle for 5 min. *Right panel*: quantification of ISO induced ERK phosphorylation (pERK) normalized to total ERK. The data represent the mean ± SEM of three independent experiments. *A* and *B*, blots are representative of three independent experiments. *C*, effect of Gα_s_ and β-arrestin1/2 knockdown on ERK activation. HeLa cells were transfected with *GNAS*, *ARRB1*, and *ARRB2* or control siRNAs for 72 h and stimulated with 1 μM ISO or vehicle for 5 min. *Left panel*: Western blot showing Gα_s_ and β-arrestin1/2 expression. HSP90 was used as loading control. *Middle panel*: representative blots of pERK and total ERK. *Right panel*: quantification of ISO induced pERK normalized to total ERK. The data represent the mean ± SEM of four independent experiments. *D*, effect of Gα_s_ and β-arrestin1/2 knockdown on β2AR target gene expression. HeLa cells were transfected with *GNAS*, *ARRB1*, and *ARRB2* or control siRNAs for 72 h and stimulated with 1 μM ISO or vehicle for 1 h. mRNA levels of PCK1 and DUSP1 genes were determined by qPCR as described in the Experimental procedures section. The data represent the mean ± SEM of at least three independent experiments. β2AR, β2-adrenergic receptor; ERK, extracellular signal–regulated kinase; ISO, isoproterenol; qPCR, quantitative PCR.

Most studies examining the relative contribution of G proteins and β-arrestins to GPCR signaling using gene-editing approaches have taken advantage of cellular systems overexpressing β2AR (15, 16, 17). The possibility still exists that the limited G protein–independent β-arrestin-mediated signaling may be conditioned by the cell context, the expression levels of the receptors, or the possible rewiring of signaling pathways in G protein and β-arrestin knockout cells (18, 46, 47). Thus, to test this possibility, we used an alternative cellular system expressing β2AR endogenously, taking advantage that HeLa cells, one of the most widely used human cell lines, express functional βARs (48, 49). In agreement with these findings, HeLa cells showed ERK phosphorylation in response to isoproterenol stimulation, and pretreatment with propranolol, a nonselective βAR antagonist, abolished the response (Fig. 7*A*). To assess the contributions of the individual βAR subtypes, we used CGP 20712 (CGP) and ICI 118,551 (ICI) to selectively block β1AR or β2AR, respectively (50). Isoproterenol-induced ERK phosphorylation was abolished by ICI but not affected by CGP, supporting that ERK activation in HeLa cells is mostly mediated by β2AR (Fig. 7*B*).

We next assessed Gα_s_ and β-arrestin roles in β2AR-mediated ERK activation (Fig. 7*C*). Knockdown of Gα_s_ abrogated ERK activation, whereas knockdown of β-arrestin1/2 did not decrease ERK phosphorylation but instead increased ERK activation in response to β2AR stimulation when compared with control siRNA (Fig. 7*C*). We also evaluated the expression of representative β2AR target genes under the same conditions (Fig. 7*D*). Knockdown of β-arrestin1/2 did not diminish the mRNA levels of PCK1 and DUSP1, whereas silencing Gα_s_ largely impaired isoproterenol-induced expression of these genes (Fig. 7*D*). These results indicate that in a cellular context expressing endogenous receptor levels, Gα_s_ protein predominates over β-arrestin1/2 to initiate β2AR-mediated ERK activation and gene expression upon isoproterenol stimulation.

**Figure 8:**
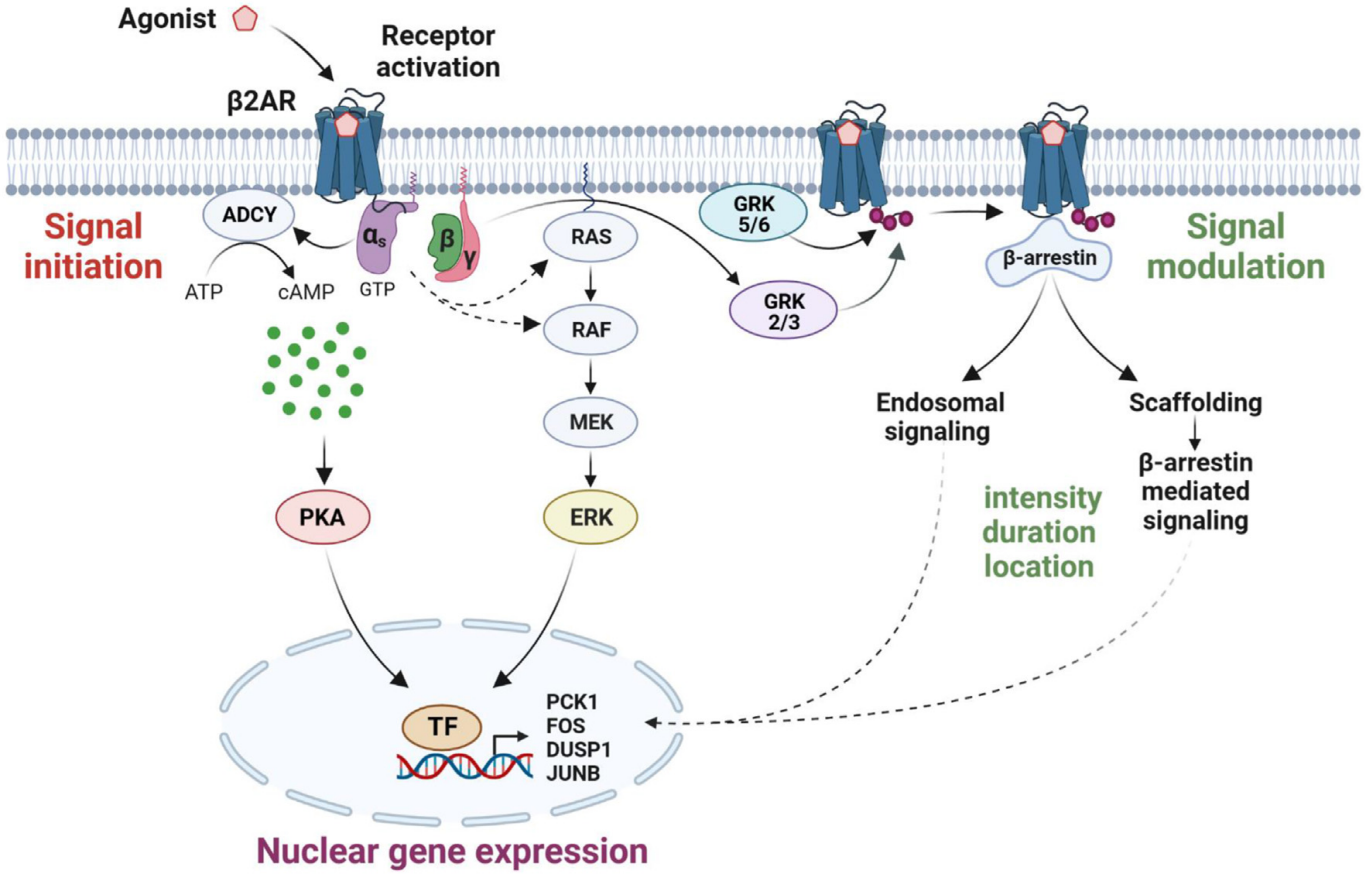
**Scheme depicting β2AR-mediated transcriptional response.** β2AR signaling is initiated by GTP-Gα_s_ and Gβγ subunits capable of activating PKA and MAPK pathways that lead to nuclear gene expression. Gs protein also dictates the GRK isoforms involved in β-arrestin recruitment. β-arrestins drive receptor desensitization and internalization. They also play a key role in endosomal signaling, serve as scaffolds for signaling elements, and may initiate their own signaling events. Altogether, β-arrestins govern intensity, duration, and location of the signal, thereby modulating the ultimate transcriptional outcome. β2AR, β2-adrenergic receptor; GRK, G protein–coupled receptor kinase; MAPK, mitogen-activated protein kinase.

Collectively, these findings support a model in which β2AR initiates signaling events downstream from Gs resulting in the nuclear expression of PKA- and ERK-regulated genes in a converging fashion, whereas β-arrestins modulate these signals because of their multiple functional roles while activating β-arrestin-specific pathways and initiating endosomal signaling, as depicted in Figure 8.

## Discussion

While it is widely accepted that heterotrimeric G proteins play a key role in transducing signals emanating from agonist-activated GPCRs, most GPCRs also concomitantly recruit and promote conformational changes in β-arrestins, which acquire an active state (6). The latter promotes receptor uncoupling from G proteins and internalization, as part of a desensitization process (12). In this context, β-arrestins may also mediate the activation of specific signaling cascades, including ERK, in parallel to or independent of G protein–mediated events (11, 12). β-arrestin signaling role has been extensively investigated, often using β2AR as a typical example of receptors coupled to G proteins and β-arrestins (13, 14). Importantly, several ligands for β2AR receptors are widely used therapeutic agents, some of which have been reported as “arrestin biased” (51, 52, 53). However, these initial observations have been recently reexamined using gene-editing approaches, thereby enabling the analysis of GPCR signaling in the absence of G proteins or β-arrestins. The emerging perspective is that β-arrestins are not strictly required for GPCR signaling to ERK, albeit playing a modulatory role in a GPCR and cell-specific fashion (15, 16, 17, 18, 47, 54). Unlike arrestin dependence, G protein independence of signals downstream of β2AR has not been investigated extensively, including the possibility that G protein deficiency may restrict β-arrestin recruitment and activation. This potential interdependence may compromise the ability to interpret the sole contribution of G proteins to β2AR signaling using KO cells. Using BRET assays and recently developed β-arrestin conformational sensors, we now show that in the absence of Gα_s_, activation of β2AR induces the recruitment and conformational changes in β-arrestin, the latter being indistinguishable from those observed in the presence of this G protein. However, β2AR-mediated β-arrestin engagement and acquisition of an active conformation failed to promote PKA and ERK nuclear gene expression programs in the absence of Gα_s_. These findings provide further support to the emerging concept that G proteins are central for Gs-GPCR signaling initiation to PKA and ERK, whereas β-arrestins play a distinct signaling role, including signal modulation once initiated through G proteins.

Our findings also support that while Gα_s_ is predominant, Gα_i_ protein is not required for β2AR signaling. As β2AR can couple (55) and signal (56) through Gα_i_ protein, it is possible that β2AR–Gα_i_ coupling may not be efficient enough to trigger detectable ERK signaling in HEK293 cells. In this sense, β2AR–Gα_i_ complexes may display weak interactions (57), with lower potency compared with Gα_s_ (55, 58). Nonetheless, Gα_i_ signaling may be relevant for β2AR responses in other cellular contexts such as cardiomyocytes (59).

An interesting finding from our studies is that Gα_s_ dictates the specificity of the individual GRKs involved in β2AR internalization and β-arrestin recruitment, which can be likely explained by the distinct GRK localization and mechanism of engagement. GRK2/3 are preferentially localized to the cytosol, whereas GRK5/6 are mostly associated with the PM (38, 60). Both GRK families phosphorylate β2AR after receptor activation, primarily in its residues S355, S356, T360, and S364. To do so, GRK2/3 are recruited to the PM through a direct interaction of Gβγ dimers with the C-terminal domain of GRK2/3 (61). GRK5/6 are instead localized to the PM by their C-terminal palmitoylation (GRK6) or interaction of polybasic regions with negatively charged phospholipids (GRK5) (60). Quadruple GRK2/3/5/6 KO cells lack any β2AR internalization and β-arrestin recruitment, but both classes of GRKs can contribute to this process as judged by (1) the partial effects observed on their respective GRK class KO cells (*i.e.*, double GRK2/3 and GRK5/6 KO cells) as well as (2) the partial rescue by GRK2 and GRK6 re-expression in GRK2/3/5/6-deficient cells. However, in the absence of Gα_s_, β2AR may not be able to activate Gs and release Gβγ dimers to recruit GRK2/3, hence only GRK5/6 are expected to remain functional.

We have addressed this possibility by performing direct measurements of GRK recruitment to β2AR and receptor phosphorylation upon isoproterenol stimulation. Indeed, our findings suggest that conformational changes in β2AR after ligand binding may expose its C-terminal phosphoacceptor sites leading to substrate recognition by GRK5/6, independently of Gs and hence receptor signaling, whereas GRK2/3 requires coupling to Gαs to recruit these GRKs to the proximity of activated β2AR. In this case, we can speculate that the fact that GRK2/3 require direct interaction with released βγ subunits from Gα protein enables these GRK isoforms to sense βγ subunit activity. As such, the absence of Gα_s_ impairs GRK2/3 recruitment likely by compromising the availability of free βγ subunits. Aligned with this possibility, a similar concept emerge from the use of GRK KO cells upon stimulation of angiotensin receptors with β-arrestin-biased agonists (37), which are expected to stimulate G protein poorly and as such, they utilize primarily GRK5/6 but not GRK2/3 for angiotensin receptor phosphorylation and internalization (37).

β-arrestin binding and activation has been originally described as a two-step process in which phosphorylated receptor C-tail engagement necessarily precedes its core engagement (62), but recent studies indicate that receptor-core and C-tail engagement can each independently mediate β-arrestin activation (63, 64, 65). Multiple types of receptor–β-arrestin complexes probably exist, with distinct or complementary functional outcomes (6, 34). We observed an increase in β-arrestin recruitment in the absence of Gα_s_ but no differences in the β-arrestin conformational changes. This suggests that in Gα_s_-deficient cells, β-arrestin may have increased access to the β2AR core and subsequent association of β-arrestin to the β2AR-phosphorylated C-tail. Thus, it is possible that in wildtype cells, Gα_s_ protein dissociation may precede the interaction of β-arrestin with the β2AR core. This possibility and the functional consequences of β-arrestin association to the receptor core *in vivo* warrant further investigation.

Our current study may have interesting implications regarding ongoing studies on the interplay between PM and endosomal signaling by β2AR. Previous reports examined Gα_s_ protein–mediated signaling in the endosomes and found that the location of cAMP production is critical in the transcriptional response mediated by β2AR (44). To prevent receptor endocytosis, these studies used pharmacological tools that inhibit dynamin or silence clathrin heavy chain. However, in these studies, β-arrestins are still able to desensitize β2AR signaling triggered at the PM. This may have resulted in reduced signaling capacity, whereas in the absence of β-arrestin1/2, β2AR endocytosis is prevented, but β2AR signaling triggered at the PM is not desensitized. Similarly, an interesting finding in our current study was the limited impact on the overall regulation of ERK-related nuclear gene expression signatures in β-arrestin KO cells with respect to wildtype cells. This is in contrast to Gα_s_ gene deletion, which nearly abolished all nuclear gene expression regulations, which can be rescued by Gα_s_ re-expression as shown for representative genes. This raises questions about the ultimate impact of the balance of ERK activation in the cytosol *versus* endosomes (66), with the latter strictly requiring β-arrestin function, as revealed by elegant single-cell studies (67). Although cytosolic ERK activation was not investigated in detail in this recent study (67), we can speculate that overall ERK activation by the stimulation of β2AR at the PM may be able to promote downstream nuclear signaling events. This possibility is further supported by the use of β2AR-C tail mutants that cannot be phosphorylated by GRKs (β2AR 3S mutant) and hence cannot recruit β-arrestin and remain at the PM after agonist stimulation (30, 36). This β2AR-C tail mutant can still activate ERK (as we previously reported (15)) and promote nuclear gene expression potently. We posit that both membrane and endosomal signaling may act in parallel, and that the increased signaling capacity of β2AR at the PM in the absence of β2AR desensitization may compensate for reduced signaling from endosomes. Ultimately, the balance between cytosolic and endosomal cAMP- and ERK-mediated signaling activated by β2AR may compensate for each other, thus promoting similar nuclear responses.

In our current study, we have also extended our observations to a cellular system expressing endogenous levels of β2AR. In HeLa cells, ERK phosphorylation triggered by β2AR resulted in a rapid response, and while knockdown of Gα_s_ abrogated ERK activation and expression of β2AR target genes, silencing β-arrestins had limited impact in these responses. Similar to HEK293 cells (15, 16), our findings in HeLa cells support that Gα_s_ protein plays a more prominent role than β-arrestins in β2AR-mediated ERK activation and expression of genes upon β2AR activation. Overall, our findings provide further support of the key role of G proteins in signaling initiation by Gs-linked GPCRs to PKA and ERK, and that β-arrestins may control additional signaling events while playing a more specific role in signal modulation, either dictating the duration of the signals or the cellular location in which the G protein–initiated signals are transmitted. Ultimately, we can speculate that the balance between these two divergent arms, G proteins and β-arrestins, acting immediately downstream of GPCR activation may govern the nuclear transcriptional responses and the biological outcomes initiated by GPCR-regulated signaling networks.

## Experimental procedures

### Reagents

(−)-Isoproterenol hydrochloride (I6504), ICI 118,551 hydrochloride (I127), PTX (P7208), and dimethyl sulfoxide (D8418) were purchased from Sigma. CGP 20712 dihydrochloride (10-241-0) and CNO (4936) were purchased from Tocris Bioscience. Propranolol hydrochloride (P0995) was obtained from Tokyo Chemical Industry. Pooled siRNAs targeting human *GNAS* (L-010825-00), *ARRB1* (L-011971-00), *ARRB2* (L-007292-00), and nontargeting pool (D-001810-10) were obtained from Horizon Discovery Biosciences/Dharmacon. Antibodies against β-arrestin1/2 (D24H9) (catalog no.: 4674), GRK6 (D1A4) (catalog no.: 5878), β-tubulin (catalog no.: 2146), hemagglutinin (HA)-tagged (C29F4) phycoerythrin (PE) conjugate (catalog no.: 14904), rabbit (DA1E) isotype control PE conjugate (catalog no.: 5742), phospho-ERK1/2 (D13.14.4E) (catalog no.: 4370), ERK (catalog no.: 9102), GAPDH (14C10) (catalog no.: 2118), and HSP90 (catalog no.: 4874) were from Cell Signaling Technology. Gαs antibody (catalog no.: 371732) was obtained from Calbiochem. GRK2 (C-15) antibody (catalog no.: sc-562) was from Santa Cruz Biotechnology. FLAG antibody (M1) (catalog no.: F-3040) was obtained from Sigma. Antibodies used to detect β2AR phosphorylation, pT360/pS364 (7TM0029B) and pS364 (7TM0029F), were obtained from 7TM Antibodies. Secondary horseradish peroxidase–conjugated antibodies (catalog nos.: 4010-05 and 1010-05) were purchased from SouthernBiotech. Alexa Fluor 647 dye was from Life Technologies, and SNAP surface Alexa Fluor 488 (catalog no.: S9129S) label was purchased from New England Biolabs.

### Plasmids and constructs

The pcDNA 3.1 HA-β2AR and Gα_s_ protein (short and long isoforms) plasmids were obtained from the complementary DNA (cDNA) Resource Center (www.cDNA.org). FLAG-β2AR wildtype and 3S (S355G, S356G, and S364G) mutant plasmids were prepared as previously described (30). The pSNAPf β2AR plasmid (N9184S) used for imaging studies was purchased from New England Biolabs. The plasmids coding for β2AR-RLucII and rGFP-CAAX were described previously (23). The RLucI-β-arrestin2 plasmid was a gift from Dr Stefano Marullo, Université de Paris, France. The β-arrestin1-RLucI plasmid was previously described (68). The peGFP-N1 Nb80 and clathrin light chain–dsRed plasmids used for TIRF microscopy were previously described (30, 69). The β-arrestin2–FlAsH–NanoLuc biosensors were prepared as previously described (34). The pCAGGS GRK2 and GRK6 plasmids were previously described (37).

To generate a doxycycline-inducible PKA Cα lentiviral construct, cDNA for wildtype murine PKA Cα was PCR amplified and recombined into the pDONR221 backbone (catalog no.: 12536017; ThermoFisher) using BP Clonase II according to the manufacturer's instructions (catalog no.: 11789020; Invitrogen). After confirming proper gene insertion with diagnostic digests, the PKA Cα cDNA was then transferred to the final pLVX TetOn 3xFLAG puro destination vector (70) with the LR clonase (catalog no.: 11791100; Invitrogen). Cα construct was tagged with a C-terminal 3xFLAG.

For the NanoBiT-GRK recruitment assay, the ssHA-FLAG-β2AR-SmBiT construct contained human full-length β2AR with the N-terminal fusion of the HA-derived signal sequence (ssHA), FLAG epitope tag, a flexible linker (MKTIIALSYIFCLVFADYKDDDDKGGSGGGGSGGSSSGGG; the FLAG epitope tag is underlined), and the C-terminal fusion of the small luciferase fragment (SmBiT). The GRK-LgBiT constructs contained human full-length GRK (GRK3 or GRK6) with the C-terminal fusion of the large fragment (LgBiT) as described previously (37). ssHA-FLAG-β2AR-SmBiT, GRK-LgBiT, and untagged human Gα_s_ (short isoform) were inserted into the pCAGGS expression vector.

### Cell culture

HEK293 wildtype, β-arrestin1/2 KO, Gαs KO, GRK2/3 KO, GRK5/6, and GRK2/3/5/6 KO cells generated using CRISPR–Cas9 technology were described previously (15, 22, 37). HEK293T cells (CRL-3216) used to prepare lentivirus and HeLa cells (CCL-2) were purchased from the American Type Culture Collection. All HEK293 cells, the engineered derivatives, and HeLa cells were cultured in Dulbecco’s modified Eagle’s medium (DMEM) (catalog no.: D6429; Sigma) supplemented with 10% fetal bovine serum (FBS) (catalog no.: F2442; Sigma) and 1% penicillin/streptomycin (catalog no.: A5955; Sigma).

### Transfection, RNA interference, and viral transduction

Transient transfections of plasmid DNAs were performed using TurboFect reagent (catalog no.: R0531; Invitrogen) for 48 h unless otherwise indicated. Transfections of siRNAs were performed using Lipofectamine RNAiMAX (catalog no.: 13778; Invitrogen) using 50 nM *ARRB1* and *ARRB2* siRNAs or 20 nM *GNAS* siRNAs for 72 h to achieve RNA interference–mediated knockdown. Cells transfected with both siRNA and plasmid DNA were first transfected with siRNA for 24 h, followed by a medium change before transfection with plasmid DNA for another 48 h.

PKA Cα lentiviruses were prepared by transfecting HEK293T cells with pLVX TetOn PKA Cα 3xFLAG, psPAX2, and VSVg plasmids in a 3:2:1 ratio using TurboFect reagent and collecting 48 and 72 h viral supernatants. FLAG-β2AR, FLAG-Gα_s_ DREADD, and FLAG-Gα_i_ DREADD lentiviruses were prepared following the same protocol but transfecting HEK293T cells with pLESIP FLAG-β2AR, pLESIP FLAG-Gα_s_-DREADD, and pLESIP FLAG-Gα_i_-DREADD lentivirus, respectively. HEK293 wildtype, GRK KO, and Gα_s_ KO cells were transduced by infection with the corresponding 0.45 mm polyvinylidene difluoride–filtered lentivirus with polybrene (8 μg/ml) and then selected with puromycin (1 μg/ml) (ant-pr-1; InvivoGen) to generate stable cell lines (HEK293 PKA Cα, HEK293wt FLAG-β2AR, GRK KO FLAG-β2AR, Gα_s_ KO FLAG-β2AR, HEK293wt FLAG-Gα_s_-DREADD, and HEK293wt FLAG-Gα_i_-DREADD). PKA Cα expression and function induced by 1 μg/ml doxycycline for 48 h was confirmed by Western blotting for FLAG, Cα, phosphoPKA substrates, and by quantitative PCR (qPCR) for target genes. FLAG-β2AR expression was verified by immunoprecipitation with FLAG beads followed by Western blotting for FLAG.

### Confocal microscopy

For experiments examining SNAP-β2AR trafficking, HEK293 wildtype, β-arrestin1/2 KO, and Gαs KO cells were seeded in polylysine-coated 18 mm glass coverslips (catalog no.: 72222-01; Fisher) in a 12-well plate and the following day transfected with pSNAPf-β2AR. After 24 h, the cells were labeled with 5 μM SNAP surface Alexa Fluor 488 dye (catalog no.: S9124S; New England Biolabs) for 30 min at 37 °C, washed three times with complete medium, and stimulated with 10 μM isoproterenol for 10 min. The cells were washed with PBS and fixed with 2% formaldehyde/PBS solution (catalog no.: 157-8; Electron Microscopy Sciences) for 15 min. Coverslips were washed three times with PBS for 5 min and mounted onto glass slides (catalog no.: P4981-001; Fisher) with ProLong Gold antifade reagent (catalog no.: P36930; Invitrogen). Confocal immunofluorescence images were collected on a Zeiss LSM 700 laser scanning microscope with 40× oil immersion lens using for Alexa Fluor 488, 488 nm excitation and 505 to 530 nm emission band-pass filter sets.

### TIRF microscopy imaging

TIRF microscopy was performed at 37 °C using a Nikon Ti-E inverted microscope equipped for through-the-objective TIRF microscopy and outfitted with a temperature-, humidity-, and CO_2_-controlled chamber (Okolab). Images were obtained with an Apo TIRF 100×, 1.49 numerical aperture objective (Nikon) with solid-state 488, 561, and 647 nm lasers (Keysight Technologies). An Andor iXon DU897 EMCCD camera controlled by NIS-Elements 4.1 software was used to acquire image sequences every 2 s for 10 min. HEK293 wildtype and Gαs KO cells were transfected with FLAG-β2AR, peGFP-N1 Nb80, and clathrin light chain–dsRed plasmids. The following day, the cells were plated on poly-l-lysine (Sigma)–coated 35 mm glass-bottomed culture dishes (MatTek Corporation). After 48 h of transfection, cells were labeled with FLAG antibody conjugated to Alexa Fluor 647 dye (Life Technologies) for 10 min at 37 °C, washed, and imaged live in DMEM without phenol red supplemented with 30 mM Hepes, pH 7.4 (University of California San Francisco Cell Culture Facility). Cells were treated with bath application of 10 μM isoproterenol at time 0 s for time course. Quantitative image analysis was performed on unprocessed images using Fiji software (71). To quantify the change in β2AR activation over time through TIRF microscopy images, Nb80 PM fluorescence was measured over the entire time series in a region of interest corresponding to the cell. Fluorescence values of the region of interest were normalized to initial fluorescence values before agonist addition. Minimal bleed-through and photobleaching were verified using single-labeled and untreated samples, respectively. At least three independent experiments were performed for live-cell TIRF microscopy imaging.

### Western blotting

#### Ligand stimulation and pharmacological treatment

For ERK activation experiments, HeLa cells seeded in 6-well plates were serum starved overnight and incubated with vehicle or isoproterenol at the concentrations and time points indicated in the figure legends. For pretreatments with antagonists, 10 μM propranolol, 300 nM CGP 20712, or 300 nM ICI 118551 were added to the culture medium 30 min before isoproterenol stimulation. HEK293 wildtype transiently transfected with β2AR, Gα_s_-DREADD, and Gα_i_-DREADD cells seeded in 6-well plates were pretreated with dimethyl sulfoxide or PTX 100 ng/ml for 18 h in serum-free media before 1 μM isoproterenol or CNO stimulation.

#### Sample preparation and immunoblotting

Cells were washed once with PBS and lysed on ice in radioimmunoprecipitation assay (RIPA) buffer (catalog no.: 9806; Cell Signaling Technology) with protease and phosphatase inhibitors (catalog nos.: B14001 and B15001; Bimake). Lysates were sonicated and cleared by centrifugation. Protein concentration in the supernatants was determined by the DC Protein Assay Kit (BioRad; catalog no.: 5000112), and equal amounts of proteins were denatured by boiling in Laemmli sample buffer (catalog no.: 1610747; Bio-Rad). Samples were then resolved on SDS-polyacrylamide electrophoresis gels and transferred to polyvinylidene difluoride membranes (catalog no.: IPVH304F0; Millipore). Membranes were blocked and probed with appropriate primary antibodies overnight at 4 °C. After incubation for 1 h at room temperature with the secondary horseradish peroxidase–conjugated antibodies, reaction products were developed with Pierce ECL Western Blotting Substrate (catalog no.: 32106; Thermo Scientific).

### β2AR phosphorylation assay

HEK293 wildtype, GRKs, and Gα_s_ KO cells stably expressing FLAG-tagged β2AR were seeded in 6-well plates. The following day, the cells were serum starved overnight. On the day of the experiment, the cells were incubated with 10 μM isoproterenol or vehicle for 10 min, washed once with PBS, and lysed on ice in RIPA buffer (catalog no.: 9806; Cell Signaling Technology) with 0.1% (w/v) SDS and protease and phosphatase inhibitors (catalog nos.: B14001 and B15001; Bimake). Lysates were sonicated and cleared by centrifugation. Protein concentration in the supernatants was determined by the DC Protein Assay Kit. FLAG beads (catalog no.: F2426; Sigma) were added to equal amounts of proteins from the supernatants and gently incubated at 4 °C on a turning wheel for 2 h. The beads were then washed four times with RIPA buffer. Proteins were eluted from the beads using Laemmli sample buffer (catalog no.: 1610747; Bio-Rad) for 30 min at 50 °C. Samples were further processed as described in the Western blotting section using the appropriate primary antibodies listed in the Reagents section.

### RNA-Seq and analysis

#### Sample preparation

To determine the role of Gαs protein and β-arrestin1/2 in β2AR transcriptional response, HEK293 wildtype, β-arrestin1/2 KO, and Gαs KO cells were seeded in poly-d-lysine-coated 6-well plates and transfected with HA-β2AR plasmid. The following day, the cells were serum starved overnight. After 48 h of transfection, the cells were incubated with 1 μM isoproterenol or vehicle for 1 h. To obtain the PKA signature, HEK293 wildtype and PKA Cα cells were seeded in poly-d-lysine-coated 6-well plates and treated with 1 μg/ml doxycycline for 48 h. After isoproterenol stimulus or doxycycline incubation, the cells were washed with PBS, total RNA was isolated with an RNeasy Mini Kit (catalog no.: 74104; Qiagen) including an on-column DNase I digestion and quantified using a Nanodrop ND-1000 (Thermo Scientific). Library preparation and paired-end 150 bp (catalog no.: PE150; Illumina) RNA-Seq was performed by Novogene Corporation.

#### Alignment/differential expression—gene set enrichment analysis

The quantification of transcripts was calculated using Salmon (version 1.7.0), which provides accurate expression estimates (72). The differential gene expression analysis including quality control, model fitting, and hypothesis testing was conducted using DESeq2 (73). To represent the strongest part of the PKA signal, our PKA signature consists of the top 100 upregulated genes. Prerank gene set enrichment analysis was performed on the DESeq2-estimated LFC values as previously described (74) using the gseapy prerank function (version 1.0.0) in Python (version 3.10.5). The MSigDB C2 collection was compared (75), and significance was assessed using 1000 permutations, and nominal *p* values less than 0.05 were considered significant.

### RT–qPCR

HEK293 wildtype and Gαs KO cells were seeded in poly-d-lysine-coated 6-well plates and transfected with siRNAs or plasmid DNAs as indicated. After 24 h of transfection with HA-β2AR plasmid, the cells were serum starved overnight. The following day, the cells were incubated with 1 μM isoproterenol or vehicle for 1 h. After washing with PBS, total RNA was isolated with an RNeasy Mini Kit (catalog no.: 74104; Qiagen) including an on-column DNase I digestion and quantified using a Nanodrop ND-1000 (Thermo Scientific). A total amount of 1 μg of RNA was reverse transcribed to cDNA using the SuperScript VILO cDNA synthesis kit (catalog no.: 11754; Invitrogen). qPCR was performed in the QuantStudio 6 Flex real-time PCR system (Applied BioSystems) using the resulting cDNA, Fast SYBR Green Master Mix (catalog no.: 4385612; Applied BioSystems) for product detection, and 400 nM of the following primers: human *PCK1* (NM_002591) forward, 5′-CTGCCCAAGATCTTCCATGT-3′ and reverse, 5′-CAGCACCCTGGAGTTCTCTC-3′; human *FOS* (NM_005252) forward, 5′-GGGGCAAGGTGGAACAGTTAT-3′ and reverse, 5′-CCGCTTGGAGTGTATCAGTCA-3′; human *DUSP1* (NM_004417) forward, 5′-AGTACCCCACTCTACGATCAGG-3′ and reverse, 5′-GAAGCGTGATACGCACTGC-3′ and human *GAPDH* (NM_001256799) forward, 5′-GAG TCA ACG GAT TTG GTC GT-3′ and reverse, 5′-TTG ATT TTG GAG GGA TCT CG-3′. The cDNA was amplified by 40 cycles of denaturing (15 s at 95 °C), annealing (30 s at 60 °C), and extension (30 s at 72 °C) steps. The specificity of each primer set was monitored by analyzing the dissociation curve, and the relative *PCK1, FOS*, and *DUSP1* expression levels were calculated by the comparative ΔΔCt method using *GAPDH* as the housekeeping gene.

### Intermolecular BRET

To study β2AR internalization and β-arrestin recruitment, HEK293 and their derived cells were seeded in 12-well plates and 24 h later transfected with 250 ng of HA-β2AR plasmid, 25 ng of a BRET donor (β2AR-RLucII or RLucI-β-arrestin), along with 250 ng of BRET acceptor (rGFP-CAAX). The following day, the cells were detached and reseeded onto poly-d-lysine-coated, white, and 96-well plates. Each transfection condition was seeded in sextuplicate for three technical replicates per treatment (isoproterenol or vehicle). After 48 h of transfection, cells were washed once with PBS, preincubated with 1.5 μM of the cell-permeable substrate methoxy e-Coelenterazine (Prolume Purple) (369; NanoLight Technology) for 10 min, and stimulated with 1 μM isoproterenol or vehicle. All BRET measurements were obtained in 30 min kinetic loop mode (11 cycles of 3 min each) using a Tecan Spark Multimode Microplate Reader, equipped with the following filters for BRET1 (center wavelength/bandwidth): 458/55 nm (donor) and 548/85 nm (acceptor), for detecting the RlucII *Renilla* luciferase (donor) and rGFP (acceptor) light emissions. Raw BRET ratio was determined by calculating the ratio of the light intensity emitted by the rGFP over the light intensity emitted by the RLucII. BRET ratios of replicates for vehicle were averaged (avgBRETvehicle). Isoproterenol-promoted BRET changes (ΔBRET) were calculated as BRETiso-avgBRETvehicle.

### Intramolecular BRET

To determine if Gαs protein was required for β-arrestin2 conformational changes, HEK293 wildtype and Gαs KO cells were seeded into 6 cm dishes and 24 h later transfected with 1.2 μg of untagged β2AR and 0.12 μg of the indicated β-arrestin–FlAsH–NanoLuc biosensor (34), according to the Effectene transfection reagent protocol (Qiagen). The following day, 40,000 cells per well were seeded into poly-d-lysine-coated 96-well plates (catalog no.: 781965; BrandTech) and incubated at 37 °C overnight. Each transfection condition was seeded in quadruplicates for three technical replicates and one mock labeling control, without the FlAsH fluorophore. On the day of the measurement, the cells were washed with PBS twice and FlAsH labeling was performed as described before (34, 76). Briefly, the cells were incubated for 1 h at 37 °C with 250 nM FlAsH in labeling buffer (150 mM NaCl, 10 mM Hepes, 25 mM KCl, 4 mM CaCl_2_, 2 mM MgCl_2_, 10 mM glucose; pH 7.3), complemented with 12.5 μM 1,2-ethane dithiol. After aspirating the FlAsH or mock labeling solutions, the cells were incubated with 250 μM 1,2-ethane dithiol in labeling buffer at 37 °C for 10 min. A 1:35,000 dilution of the NanoLuc-substrate furimazine (catalog no.: N157A; Promega) in measuring buffer (140 mM NaCl, 10 mM Hepes, 5.4 mM KCl, 2 mM CaCl_2_, 1 mM MgCl_2_; pH 7.3) was added right before the measurement of the basal values for 3 min. The cells were stimulated with different concentrations of isoproterenol, ranging from 1 nM to 10 μM, and measured for 5 more minutes. The measurements were conducted with a Synergy Neo2 plate reader (Biotek; Gen5 software), using a custom-made filter cube (excitation bandwidth 541–550 nm, emission 560–595 nm, and fluorescence filter 620/15 nm). For the initial concentration-dependent BRET changes, technical replicates were averaged and the mean of the data points after stimulation was divided by the respective mean basal values. To correct for the labeling efficiency, the mock labeling values for each transfection were subtracted. Finally, the corrected BRET change was divided by the vehicle control and calculated in percent (mean Δnet BRET changes). For the bar graphs and radar plots, the value of the highest stimulating concentrations (1 and 10 μM) was used.

### NanoBiT-GRK recruitment assay

Isoproterenol-induced GRK recruitment to β2AR was measured by the NanoBiT-based assay (37). HEK293 wildtype and Gα_s_ KO cells were seeded in a 6-well culture plate at a concentration of 2 × 10^5^ cells/ml (2 ml per well in DMEM [Nissui] supplemented with 5% FBS [Gibco], glutamine, penicillin and streptomycin), 1 day before transfection. Transfection solution was prepared by combining 6 μl (per well hereafter) of polyethylenimine Max solution (1 mg/ml; Polysciences), 200 μl of Opti-MEM (Thermo Fisher Scientific), and a plasmid mixture consisting of 500 ng ssHA-FLAG-β2AR-SmBiT and 500 ng GRK-LgBiT constructs. For the rescue experiment, 100 ng plasmid of Gα_s_ was cotransfected into the Gα_s_ KO cells with the ssHA-FLAG-β2AR-SmBiT and GRK-LgBiT plasmids. After incubation for 1 day, the transfected cells were harvested with 0.5 mM EDTA-containing Dulbecco’s PBS, centrifuged, and suspended in 2 ml of Hanks' balanced salt solution containing 0.01% bovine serum albumin (fatty acid–free grade; SERVA) and 5 mM Hepes (pH 7.4) (assay buffer). The cell suspension was dispensed in a white 96-well plate at a volume of 80 μl per well and loaded with 20 μl of 50 μM coelenterazine (Angene) diluted in the assay buffer. After a 2 h incubation at room temperature, the plate was measured for baseline luminescence (SpectraMax L; Molecular Devices), and titrated concentrations of isoproterenol ranging from 1 nM to 100 μM (Sigma–Aldrich; 20 μl; 6× of final concentrations) were manually added. The plate was immediately read for the second measurement as a kinetics mode, and luminescence counts recorded from 2.5 min to 3.5 min after compound addition were averaged and normalized to the initial counts. The fold-change values were further normalized to those of vehicle-treated samples and used to plot the GRK recruitment response.

### Flow cytometry

β2AR surface levels in HEK293 wildtype, β-arrestin1/2 KO, and Gαs KO cells were determined by HA-PE surface staining. We set up the adequate amount of β2AR plasmid necessary to transfect the HEK293 wildtype and their derived cells to achieve similar expression levels. The cells (3 × 10^5^ cells/well) were seeded in 6-well plates and transfected with 150, 500, and 1500 ng of HA-β2AR plasmid. After 48 h of transfection, the cells were detached with 10 mM EDTA in PBS and centrifuged at 1200 rpm for 3 min. Cell pellets were resuspended in PBS, transferred to V bottom 96-well plates, and incubated 20 min in the dark with Zombie Aqua Fixable Viability Kit (catalog no.: 423101; BioLegend) for live/dead cell discrimination. Cell suspensions were washed with fluorescence-activated cell sorting buffer (5% FBS in PBS) and stained with HA-tag-PE and isotype control-PE antibodies for 30 min at 4 °C protected from light. Stained cells were washed with fluorescence-activated cell sorting buffer and then fixed with BD Cytofix for 15 min at 4 °C, protected from light. Samples were analyzed using a BD LSRII Fortessa Cell Analyzer. Downstream analysis on live-gated cells was performed using TreeStar FlowJo software, version 10.6.2. Although we did not normalize the data to receptor levels in each experiment, under these conditions, we routinely obtain similar high transfection efficiency and level of receptor expression.

### Statistical analyses

Statistical analyses of data and best fits of BRET curves presented throughout this study were performed using GraphPad Prism 9.4.0 software (GraphPad Software, Inc). Using GraphPad Prism software, the GRK recruitment signals were fitted to a four-parameter sigmoidal concentration–response curve with a constraint of the Hill slope to absolute values less than 2. For each condition, the parameter span (= top – bottom) and pEC_50_ were used to calculate GRK responses. Band intensities of Western blots were quantified using Gel-Pro Analyzer 4.1 software (Media Cybernetics). The data were analyzed by *t* test or ANOVA test followed by Tukey’s or Dunnett’s post tests. The mean differences of at least three independent experiments were considered significant when *p* values were <0.05 (asterisks denote: ∗*p* < 0.05, ∗∗*p* < 0.01, ∗∗∗*p*< 0.001, and ∗∗∗∗*p*< 0.0001).

## Data availability

All data associated with this study are presented within the article. RNA-seq files have been deposited to NCBI's Gene Expression Omnibus under GEO Series accession numbers: GSE245270 for the ?2AR transcriptional response and GSE245789 for the PKA signature. Further information and requests for resources and reagents should be directed to and will be fulfilled by the lead contact, Dr J. Silvio Gutkind (sgutkind@health.ucsd.edu).

## Supporting information

This article contains supporting information.

## Conflict of interest

J. S. G. is consultant for Domain Therapeutics, Pangea Therapeutics, and io9, and founder of Kadima Pharmaceuticals, outside the submitted work. M. B. is the president of Domain Therapeutics Scientific Advisory Board. The authors declare that they have no conflicts of interest with the contents of this article.
