# Supplementary material for: Gαs is dispensable for β-arrestin coupling but dictates GRK selectivity and is predominant for gene expression regulation by β2-adrenergic receptor
Source: J Biol Chem. 2023 Sep 27;299(11):105293. doi: 10.1016/j.jbc.2023.105293 (PMC10641165; doi:10.1016/j.jbc.2023.105293)
Supplement: Supporting information [file mmc5.pdf]

Supporting information for

Gαs is dispensable for β-arrestin coupling but dictates GRK selectivity and is predominant for gene expression regulation by β2-adrenergic receptor

Valeria Burghi, Justine S Paradis, Adam Officer, Sendi Adame Garcia, Xingyu Wu, Edda SF Matthees, Benjamin Barsi-Rhyne, Dana J Ramms, Lauren Clubb, Monica Acosta, Pablo Tamayo, Michel Bouvier, Asuka Inoue, Mark von Zastrow, Carsten Hoffmann, J Silvio Gutkind *

*Correspondence: sgutkind@health.ucsd.edu

The PDF file includes:

Fig. S1. Surface HA-β2AR abundance in HEK293 wild type, β-arrestin1/2 KO and Gαs KO cells.
Fig. S2. ERK activation after $G\alpha_i$ protein inhibition.
Fig. S3. Validation of the expression levels of β2AR target genes in the absence of β-arrestin1/2 or in the presence of the phosphorylation-deficient mutant β2AR-3S.

Other supporting information for this manuscript includes the following:

Table S1. Differentially expressed genes in HEK293 PKA Cα cells compared with HEK293 parental cells.
Table S2. Normalized counts for all genes per sample as result of RNAseq analysis for β2AR transcriptional response.
Table S3. Complete list of gene sets from the Molecular Signatures Database (MSigDB), c2 collection, computed using Gene set enrichment analysis (GSEA) for wild type, β-arrestin1/2 KO and Gαs KO cells after isoproterenol stimulation.
Table S4. Selected list of gene sets from the Molecular Signatures Database (MSigDB), c2 collection, computed using Gene set enrichment analysis (GSEA) for wild type, β-arrestin1/2 KO and Gαs KO cells after isoproterenol stimulation.

Figure S1

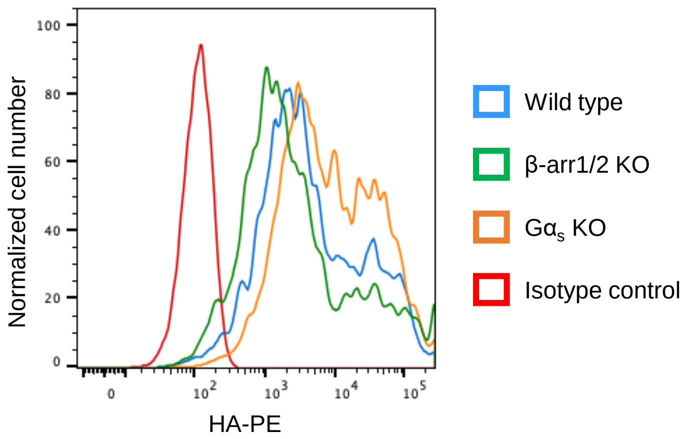

Fig. S1. Surface HA-β2AR abundance in HEK293 wild type, β-arrestin1/2 KO and $G\alpha_s$ KO cells. Flow cytometry analysis of transiently expressed HA-β2AR in HEK293 cells and engineered derivatives using HA-Tag and Isotype control antibodies. Samples were processed as described in Experimental Procedures. Results correspond to cells transfected with 500 ng/well generating signal distinguishable from isotype control and similar expression levels among cell types. Representative of three independent experiments.

Figure S2

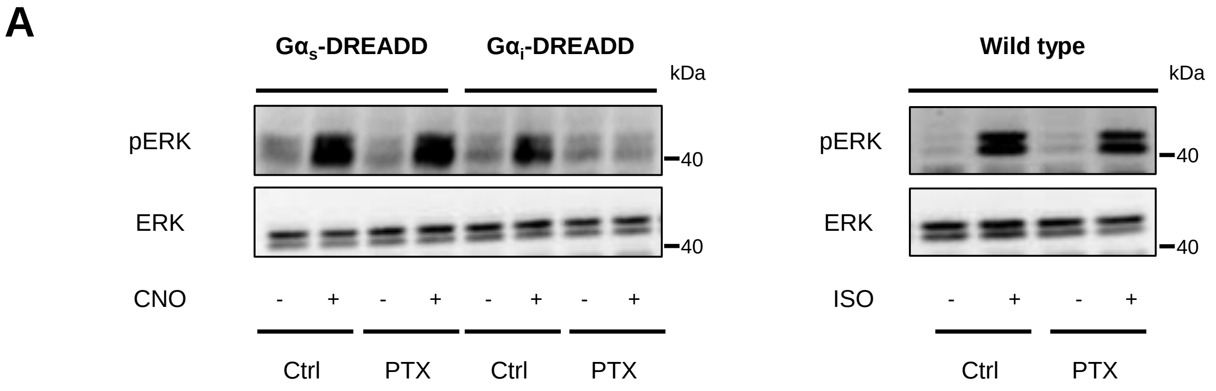

Fig. S2. ERK activation after Gα$_i$ protein inhibition. HEK293 cells stably expressing Gα$_s$ DREADD, Gα$_i$ DREADD and transiently transfected with HA-β2AR (wild type) were pretreated with DMSO (Ctrl) or Pertussis toxin (PTX) 100 ng/mL in serum free media for 18 hours. Cells were then stimulated with ISO (1 μM) or CNO (1 μM) for 5 min. Blots are representative of three independent experiments.

Figure S3

A

Silencing β-arr1/2 in wild type cells

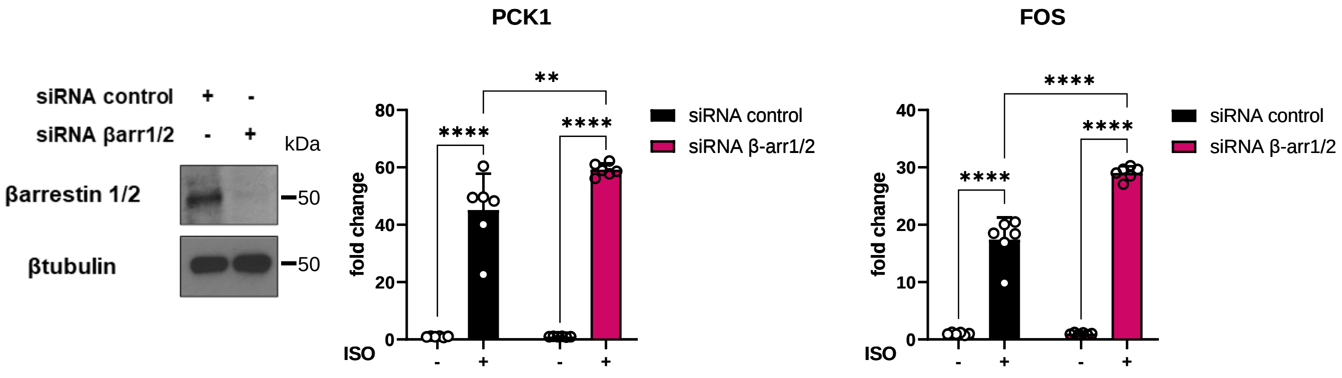

B

Wild type cells expressing β2AR-3S

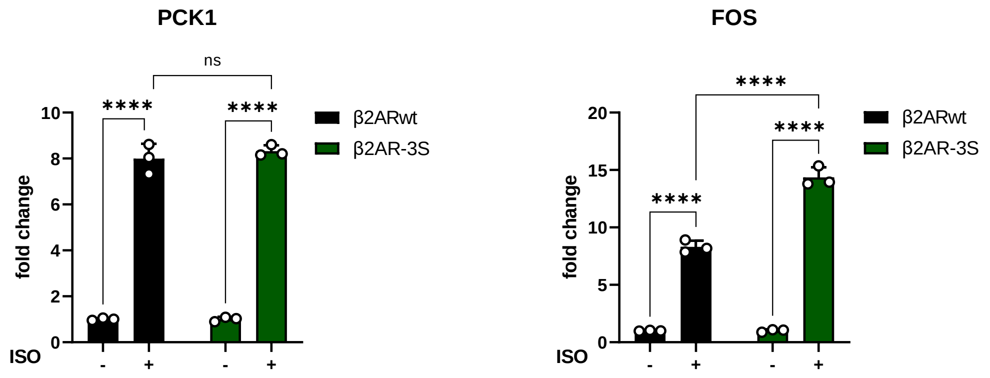

Fig. S3. Validation of the expression levels of β2AR target genes in the absence of β-arrestin1/2 or in the presence of the phosphorylation-deficient mutant β2AR-3S. Cells were incubated with 1 μM isoproterenol or vehicle for 1 h and mRNA levels of the indicated genes were determined by qPCR as described in Experimental Procedures. A) Effect of βarrestin1/2 knockdown on PCK1 and FOS genes expression in wild type cells. Cells were first transfected with *ARRB1* and *ARRB2* or control siRNAs for 24 hours, followed by a medium change before transfection with HA-β2AR for another 48 hours. Left panel: Western blot showing βarrestin1/2 expression. βtubulin was used as loading control. B) mRNA levels of PCK1 and FOS genes in HEK293 wild type cells expressing β2AR wild type or the phosphorylation-deficient mutant β2AR-3S. The data represent the mean ± s.e.m. of six (A) or three (B) independent experiments.

Table S1. Differentially expressed genes in HEK293 PKA Cα cells compared with HEK293 parental cells. Gene names, Log2FoldChange, Log2FoldChangeSE, pvalue and padj values used to generate the volcano plot of **Fig. 5B** are displayed. Our PKA signature consists of the top 100 upregulated genes.

Table S2. Normalized counts for all genes per sample as result of RNAseq analysis for β2AR transcriptional response. Normalized counts for all genes per sample, wild type, Gαs KO and β-arrestin KO cells, basal and isoproterenol stimulated conditions in triplicates as result of RNAseq analysis are displayed.

Table S3. Complete list of gene sets from the Molecular Signatures Database (MSigDB), c2 collection, computed using Gene set enrichment analysis (GSEA) for wild type, β-arrestin1/2 KO and Gα_s KO cells after isoproterenol stimulation. Gene sets names, score, normalized score, pval and FDR are displayed.

Table S4. Selected list of gene sets from the Molecular Signatures Database (MSigDB), c2 collection, computed using Gene set enrichment analysis (GSEA) for wild type, β-arrestin1/2 KO and Gα_s KO cells after isoproterenol stimulation. Gene sets related to RAS signaling, RAF signaling, PKA signaling, p38 MAPK signaling, MAPK/ERK signaling, Forskolin response and CREB signaling. Those gene sets with $p<0.05$ value for each cell line are listed separately. Their names, score, normalized score, pval and FDR are displayed. For those gene sets which were included in **Fig 5D**, leading edge genes for wild type and β-arrestin1/2 KO cells are informed.
